# Evaluation of the new TNM-staging system for thymic malignancies: impact on indication and survival

**DOI:** 10.1186/s12957-017-1283-4

**Published:** 2017-12-02

**Authors:** Michael Ried, Maria-Magdalena Eicher, Reiner Neu, Zsolt Sziklavari, Hans-Stefan Hofmann

**Affiliations:** 1 Department of Thoracic Surgery, University Medical Center Regensburg, Franz-Josef-Strauß-Allee 11, 93053 Regensburg, Germany; 2 Department of Thoracic Surgery, Hospital Barmherzige Brüder Regensburg, Regensburg, Germany

**Keywords:** Thymoma, Thymic carcinoma, TNM staging, Masaoka-Koga, Staging system

## Abstract

**Background:**

The objective of this study is the evaluation of the Masaoka-Koga and the International Association for the Study of Lung Cancer (IASLC)/International Thymic Malignancy Interest Group (ITMIG) proposal for the new TNM-staging system on clinical implementation and prognosis of thymic malignancies.

**Methods:**

A retrospective study of 76 patients who underwent surgery between January 2005 and December 2015 for thymoma. Kaplan–Meier survival analysis was used to determine overall and recurrence-free survival rates.

**Results:**

Indication for surgery was primary mediastinal tumor (*n* = 55), pleural manifestation (*n* = 17), or mediastinal recurrence (*n* = 4) after surgery for thymoma. Early Masaoka-Koga stages I (*n* = 9) and II (*n* = 14) shifted to the new stage I (*n* = 23). Advanced stages III (Masaoka-Koga: *n* = 20; ITMIG/IASLC: *n* = 17) and IV (Masaoka-Koga: *n* = 33; ITMIG/IASLC: *n* = 35) remained nearly similar and were associated with higher levels of WHO stages. Within each staging system, the survival curves differed significantly with the best 5-year survival in early stages I and II (91%). Survival for stage IV (70 to 77%) was significantly better compared to stage III (49 to 54%). Early stages had a significant longer recurrence-free survival (86 to 90%) than advanced stages III and IV (55 to 56%).

**Conclusions:**

The proportion of patients with IASLC/ITMIG stage I increased remarkably, whereas the distribution in advanced stages III and IV was nearly similar. The new TNM-staging system presents a clinically useful and applicable system, which can be used for indication, stage-adapted therapy, and prediction of prognosis for overall and recurrence-free survival.

## Background

Main objective of therapy in all stages of thymoma and thymic carcinoma should be complete surgical resection based on a stage-adapted treatment including multimodality therapy in advanced stages [1–3]. Prognosis depends on the preoperative tumor stage mainly characterized by the anatomical extend (Masaoka-Koga classification), the World Health Organization (WHO) histological classification system, and the completeness of surgical resection [4–8]. At least 15 different stage classification systems for thymic tumors have been proposed and implemented in the last decades [9]. However, the Masaoka-Koga classification remains the most common and frequently applied clinical staging system [10].

Recently, the International Association for the Study of Lung Cancer (IASLC) and the International Thymic Malignancy Interest Group (ITMIG) have proposed a new classification for thymic malignancies [11, 12]. The IACLC/ITMIG staging system has now been approved by the Union for the International Cancer Control (UICC) and the American Joint Committee on Cancer (AJCC) in the eight edition of the TNM classification [13, 14].

Objective of this study was the evaluation of patients with thymoma or thymic carcinoma who underwent surgical resection and to compare their characteristics, indications for surgery, and outcomes when classified according to the Masaoka-Koga system and with the new proposed IASLC/ITMIG TNM-staging system.

## Methods

### Study design

This was a retrospective, non-randomized study at the Department of Thoracic Surgery, University Medical Center Regensburg, and the Department of Thoracic Surgery, Hospital Barmherzige Brüder Regensburg. Between January 2005 and December 2015, a total of 76 consecutive patients with thymoma or thymic carcinoma, who underwent radical surgical resection, were included.

The study was approved by our Institutional Review Board, which waived the requirement for an individual patient consent because only routine patient data were used for this retrospective analysis. Patient characteristics and operative reports were obtained from the institutional database and medical records. Preoperative staging included contrast-enhanced computed tomography (CT) scan of the chest in all patients. The preoperative clinical and pathologic stage was determined according to Masaoka-Koga staging system [7]. The pathologic results were classified according to the WHO histological classification system [14, 15]. The proposed IASLC/ITMIG stages were determined, using the preoperative imaging in addition to histological, surgical, and medical reports.

Induction therapy was administered in patients with advanced tumor stages, which were considered not completely resectable and in order to increase the chance for complete resection. Adjuvant therapies including chemotherapy or radiotherapy were accomplished depending on tumor stage, histology, completeness of resection, and the postoperative patient’s status [3]. A further reason for not performing adjuvant therapy was patient refusal.

During the study period, eight patients with recurrence of thymoma underwent surgical resection again without any complication and with a macroscopic complete tumor resection (mediastinal or lung: R0; pleural: R0/R1).

### Proposed IASCL/ITMIG staging system

The proposed IASLC/ITMIG staging system was first published 2014 and is a new evidence-based TNM-staging system for thymic malignancies according to the TNM classification of the UICC of malignant tumors (Table 1) [11, 13, 14]. The greatest significance has the T descriptor, which evaluates the local tumor invasion, whereas nodal or distant metastases are uncommon [16]. It describes the invasion into mediastinal fat (T1a), mediastinal pleura (T1b), pericardium (T2), and other surrounding structures or organs (T3, T4) [17]. The N descriptor distinguishes between involvement of N1 (anterior/perithymic) and N2 (deep intrathoracic/cervical) lymph nodes [18]. The M descriptor indicates whether pleural and pericardial nodules (M1a) or distant organ metastases (M1b), including pulmonary intraparenchymal metastases, are evident [19].

**Table 1:** Stages of thymic tumors according to Masaoka-Koga classification and the proposed IASLC/ITMIG classification [5, 7, 13]

Stage	Masaoka-Koga	IASCL/ITMIG
I	Completely encapsulated tumor Invasion into but not through the capsule	T1N0M0 T1a: Encapsulated or extending into the anterior mediastinal fat T1b: Direct involvement of the mediastinal pleura
II	(a) Microscopic transcapsular invasion (b) Macroscopic invasion into thymic or surrounding fatty tissue Grossly adherent to but not breaking through mediastinal pleura or pericardium	T2N0M0 T2: Invasion to the pericardium
III	Invasion into neighboring organ including - Mediastinal pleura - Pericardium - Visceral pleura or lung - Phrenic or vagus nerve - Major vessels Adherence of lung or adjacent organs	(a) T3N0M0 T3: Invasion to - Lung - Brachiocephalic vein - Superior vena cava - Chest wall - Phrenic nerve (b) T4N0M0 T4: Invasion to - Aorta - Intrapericardial pulmonary artery - Myocardium - Trachea - Esophagus
IV	(a) Pleural or pericardial metastases (b) Any nodal involvement and distant metastases	(a) TanyN1M0, TanyN0M1a N1: Anterior (perithymic) nodes M1a: Separate pleural or pericardial nodule (b) N2 and/or M1b N2: Deep intrathoracic or cervical nodes M1b: Pulmonary intraparenchymal nodule or distant organ metastasis

### Follow-up

Overall survival and recurrence-free survival were calculated from the date of surgery. Follow-up analysis was conducted until either death or at the end of March 2016 in order to document tumor recurrence and the patients’ overall survival.

### Statistical analysis

Statistical analysis was performed with SPSS 16.0 (SPSS Inc., Chicago, IL) for Windows (Microsoft Corp, Redmond, WA). Descriptive statistics were used to describe patient characteristics and to compare variables. Categorical data are shown as frequency distributions (*n*) and percentages (%). The Kaplan–Meier method was used to plot survival curves, and the log-rank test was used to evaluate differences between subgroups. To perform the statistical evaluation of the two staging systems, we estimated the hazard ratio for each group in the two systems using the Cox regression analysis. Differences with a *p* value of < 0.05 were considered to be statistically significant.

## Results

### Patient characteristics

Patient characteristics are listed in Table 2. The study comprised a total of 76 patients (53.9% male) with evidence of thymoma or thymic carcinoma, which was first identified as a primary mediastinal tumor (*n* = 55; 72.4%), pleural manifestation (*n* = 17; 22.4%), or as a suspected mediastinal recurrence (*n* = 4; 5.2%) after resection of a malignant thymic tumor before the study period. Patients with advanced tumor stages (Masaoka-Koga stages III and IV) received frequently preoperative medicamentous treatment (*n* = 33; 43.4%) with induction chemotherapy (*n* = 28; 36.8%) or octreotid/prednisone (*n* = 5; 6.6%).

**Table 2:** Patient characteristics

Variable	*n* = 76 (100%)
Gender	
Male	41 (53.9)
Female	35 (46.1)
Age (mean; ±SD) [years]	52.6 (± 14.3)
Myasthenia gravis	39 (51.3)
Type of tumor	
Primary mediastinal tumor	55 (72.4)
Pleural tumor spread	17 (22.4)
Mediastinal recurrence	4 (5.2)
WHO classification	
A	2 (2.6)
AB	5 (6.6)
B1	5 (6.6)
B2	28 (36.8)
Mixed B2/B3	8 (10.5)
B3	18 (23.7)
C (thymus carcinoma)	10 (13.2)
Preoperative treatment	
None	43 (56.6)
Induction chemotherapy	28 (36.8)
Octreotid/prednisone	5 (6.6)
Radiotherapy	0 (0)

*SD* standard deviation, *WHO* World Health Organization

### Operative data

Details of the surgical procedures and postoperative data are listed in Table 3. In all patients, a radical tumor resection including the en bloc resection of the thymoma, along with the thymic gland, perithymic fat tissue, mediastinal pleura (*n* = 44; 57.9%), and pericardium (*n* = 33; 46.1%), was performed. When appropriate, the resection was extended to the lung (*n* = 32; 42.1%) or the diaphragm (*n* = 17; 22.4%). In patients with pleural tumor spread (Masaoka-Koga stage IVa: *n* = 28), tumor resection was achieved by pleurectomy/decortication (P/D: *n* = 12), extended P/D (*n* = 10), extrapleural pneumonectomy (EPP: *n* = 3), or localized pleural tumor resection (*n* = 3). An additional hyperthermic intrathoracic chemotherapy (HITHOC) with cisplatin was done in *n* = 19 patients (68%). A complete tumor resection (R0) was achieved in 48.7% of patients. A microscopic (R1: *n* = 28; 36.8%) or macroscopic (*n* = 11; 14.5%) incomplete tumor resection was documented in advanced stages III (*n* = 12) and IV (*n* = 26), due to tumor infiltration of adjacent structures or diffuse pleural manifestation. Only one stage II tumor had a R1 situation, which was treated with adjuvant radiotherapy.

**Table 3:** Operative and postoperative data

Variable	*n* = 76 (100%)
Surgery	
One approach	41 (53.9)
Two approaches	35 (46.1)
Surgical procedure	
Radical thymectomy	61 (18.3)
P/D	12 (15.8)
eP/D	10 (13.2)
EPP	3 (3.9)
HITHOC	19 (25)
Completeness of resection	
R0	37 (48.7)
R1	28 (36.8)
R2	11 (14.5)
Complications	
Intraoperative	3 (3.9)
Postoperative	10 (13.2)
Adjuvant treatment	
Chemotherapy	22 (28.9)
Radiotherapy	19 (25)
30-day mortality	0 (0)

*eP/D* extended pleurectomy/decortication, *EPP* extrapleural pneumonectomy, *HITHOC* hyperthermic intrathoracic chemotherapy, *P/D* pleurectomy/decortication, *R0* no residual tumor, *R1* microscopic residual tumor, *R2* macroscopic residual tumor

### Stage distribution

Stage distribution according to the Masaoka-Koga staging system and the proposed TNM-staging system is based on the pathological results and shown in Table 4. When comparing both classifications, Masaoka-Koga stage I patients (*n* = 9) increased to IASLC/ITMIG stage I patients (*n* = 23), and all Masaoka-Koga stage II patients (*n* = 14) decreased to *n* = 1 IASLC/ITMIG stage II patient. One patient with infiltration of the pericardium (Masaoka-Koga stage III) is now classified as IASLC/ITMIG stage II patient. The proportion of patients with advanced tumor stages III and IV was nearly similar between both classifications. The stage distribution of the new system in relation to the WHO classification is presented in Table 5. Advanced tumor stages are associated with higher levels of WHO stages (B2 to C).

**Table 4:** Patient distribution (*n*) between proposed IASLC/ITMIG stages and Masaoka-Koga classification

		IASLC/ITMIG stages						Total
		I	II	IIIa	IIIb	IVa	IVb	
Masaoka-Koga	I	9	0	0	0	0	0	9
	IIa	6	0	0	0	0	0	6
	IIb	8	0	0	0	0	0	8
	III	0	1	15	2	2	0	20
	IVa	0	0	0	0	28	0	28
	IVb	0	0	0	0	0	5	5
Total		23	1	15	2	30	5	76

**Table 5:** Patient distribution (*n*) between proposed IASLC/ITMIG stages and WHO classification

		IASLC/ITMIG stages						Total
		I	II	IIIa	IIIb	IVa	IVb	
WHO	A	2	0	0	0	0	0	2
	AB	4	0	0	0	1	0	5
	B1	2	0	1	0	2	0	5
	B2	9	1	6	0	10	2	28
	Mixed B2/B3	1	0	3	0	4	0	8
	B3	4	0	3	2	9	0	18
	C	1	0	2	0	4	3	10
Total		23	1	15	2	30	5	76

### Survival analysis

**Fig. 1:**
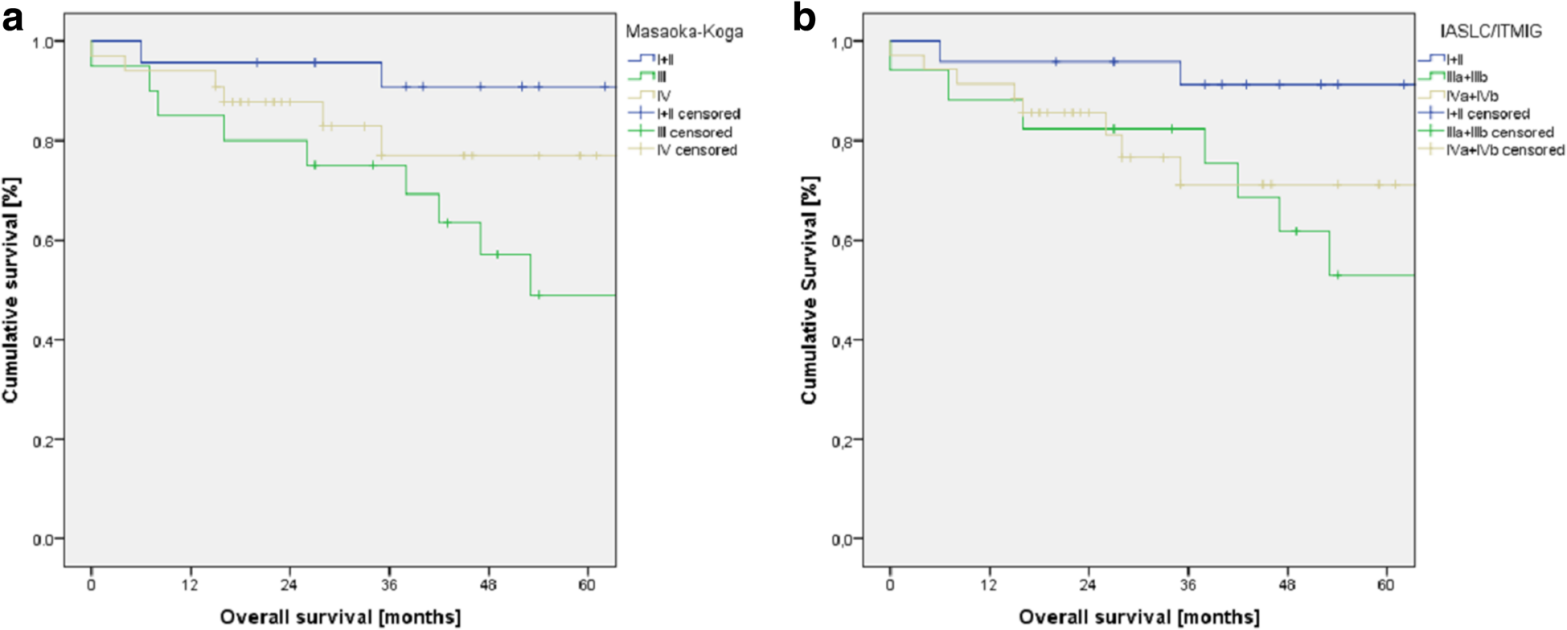
The number of patients in each subgroup is presented as well as the 5-year survival rate [%]. **a** Significant differences in survival were documented with respect to three groups of Masaoka-Koga stages. Stage III patients had a lower survival than patients with stage IV. **b** Survival curves with respect to the proposed IASLC/ITMIG stages showed also significant differences

**Fig. 2:**
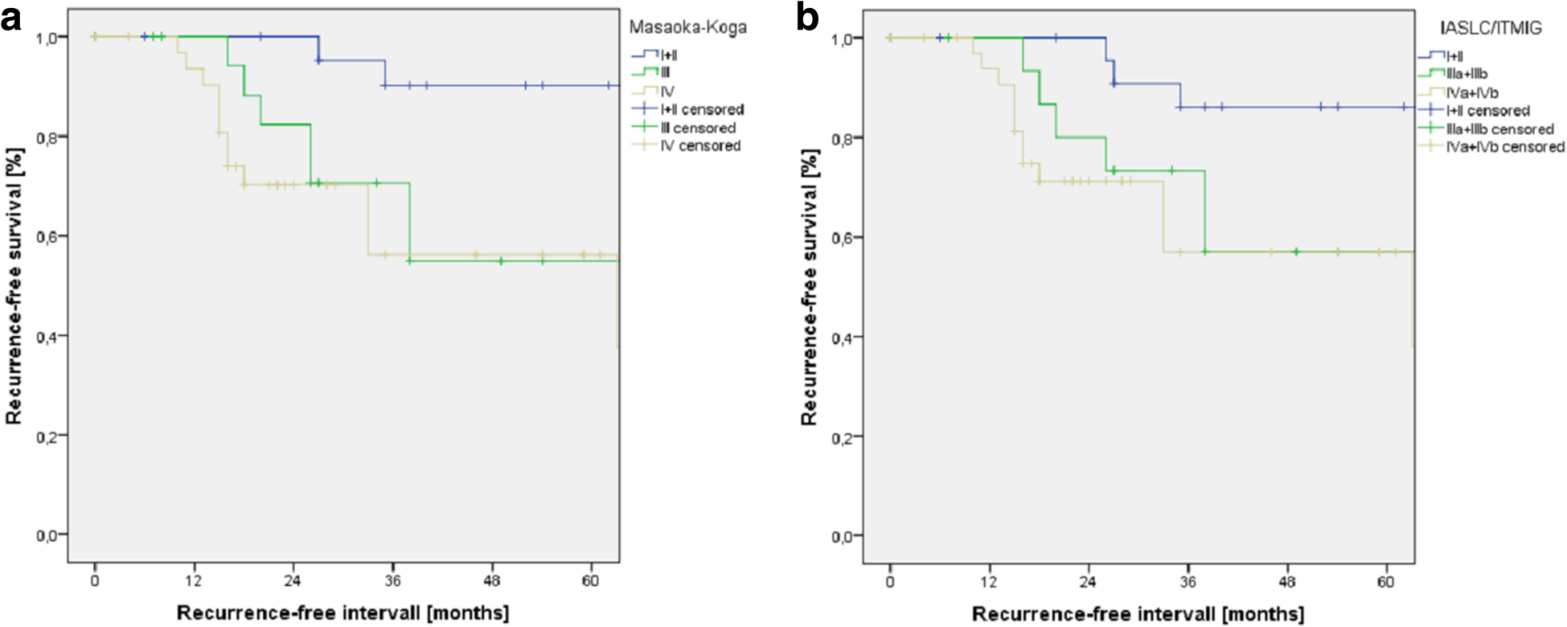
Recurrence-free survival curves for patient subgroups according to the three stage groupings. There were significant differences between stage I + II compared to stage III or stage IV with respect to the Masaoka-Koga (**a**) and the proposed IASLC/ITMIG (**b**) staging system. Recurrence-free survival was nearly similar between stage III and stage IV

Overall 5-year survival rate of all patients was 73%. Because of the small sample size if distributed in all stages, early stages I and II were joint together (*n* = 23) and compared to advanced stage III (*n* = 20) or stage IV (*n* = 33). Overall survival curves of the three subgroups according to both staging systems are displayed in Fig. 1. Within each staging system, the survival curves differed significantly. A better survival for stage IV (Masaoka-Koga 77%; IASLC/ITMIG 70%) compared to stage III (Masaoka-Koga 49%; IASLC/ITMIG 54%) was observed. Overall 5-year recurrence-free survival was 66% irrespective of stage. Early stages had a significant longer recurrence-free survival (Masaoka-Koga 90%, *p* = 0.007; IASLC/ITMIG 86%, *p* = 0.019) than advanced stages III and IV (55 to 56%), which were nearly similar (Fig. 2).

All patients with stage IVa tumors due to pleural spread received multimodality therapy and reached a 5-year survival rate up to 79%. No significant differences in survival were documented between primary and secondary (recurrence) stage IVa (*p* = 0.616).

The hazard ratios for all stages and subgroups for both staging systems using the Cox regression analysis are listed in Table 6. Overall survival in patients with Masaoka-Koga stage III (*p* = 0.02) or IASLC/ITMIG stage IIIa/IIIb (*p* = 0.03) was significantly declined. Multivariate analysis was performed including stage, resection status, and histology and showed that only the classification according to IASCL/ITMIG significantly (*p* = 0.03) influenced the overall survival.

**Table 6:** Comparison of hazard ratios between Masaoka-Koga and proposed IASLC/ITMIG stages using Cox’s regression analysis

	Overall survival			Recurrence-free survival		
	HR	95% CI	*p* value	HR	95% CI	*p* value
Masaoka-Koga						
I + II	1.00		0.054	1.00		0.03
III	6.38	1.38–29.59	0.02	5.79	1.19–28.01	0.03
IVa + IVb	3.64	0.75–17.67	0.11	8.1	1.78–36.79	0.007
IASLC/ITMIG						
I + II	1.00		0.09	1.00		0.04
IIIa + IIIb	5.71	1.19–27.49	0.03	3.66	0.91–14.67	0.07
IVa + IVb	4.73	1.01–22.1	0.048	5.31	1.47–19.15	0.01

*CI* confidence interval, *HR* hazard ratio

## Discussion

The tumor–node–metastasis (TNM) classification of cancers proposed by the UICC is used worldwide and accepted as a uniform classification system. It provides a stage-adapted indication system for therapy and helps to estimate patients’ prognosis [14]. Recently, there is a proposed TNM-staging system, which should be applicable to all types of thymic malignancies. This new system is based on a large collaborative amount of 10.808 patient data available from the ITMIG/IASLC retrospective database, leaving 8.145 of patients for final analysis. Surgery was included in 99% of these cases [11, 17].

In our study sample, the proportion of patients with the proposed IASLC/ITMIG stage I increased remarkably because they were reclassified from Masaoka-Koga stage IIa and IIb diseases. However, this changing in distribution of early-stage thymoma had no impact on indication for radical surgery.

The Masaoka-Koga stage III is heterogenous, because it includes invasion of the mediastinal pleura (T1b), pericardium (T2), and other surrounding structures (T3, T4) [20]. In the new proposed system, stage III is divided in more or most resectable stage IIIa and less or unresectable stage IIIb tumors [17]. According to that, both patients with stage IIIb in this study had invasion of the aorta or the supraaortic branches and should be considered only individually for surgical resection, since the risk of R2 resection is high [21]. Therefore, the TNM system better characterizes this heterogenous stage III and supports the indication for surgery or not surgery. Nevertheless, worst survival was registered in patients with Masaoka-Koga stage III or proposed stage IIIa/IIIb thymic tumors.

Especially in patients with advanced thymoma not eligible for immediate resection, induction therapy is recommended [22]. Surgical resection should be part of a multimodality therapy including chemotherapy and in some cases adjuvant radiotherapy to decrease the risk of recurrence and improve survival [23, 24]. These neo-adjuvant treatment indications were not influenced by the new TNM-staging system.

Furthermore, the new TNM-staging proposal also provides information on lymphatic involvement and tumor dissemination [13, 18]. Since the significance of the lymph node dissection and its impact on adjuvant therapy are still not clear, the imperative for a radical lymph node dissection remains questionable [16, 25]. So, most of all do not routinely perform a systematic lymph node dissection in all patients with thymoma and only suspicious nodes were resected for histological investigation. Therefore, we have problems with defining accurately the lymph node status [26].

The changing within early stages did not negatively affect the 5-year survival rate of 91%, because both early stages I and II include tumors, which should be completely resectable, even if they show invasion of the perithymic fat tissue, the mediastinal pleura, or the pericardium. Less imbalance of stage distribution was found in advanced stages III and IV. These results are also in line with the literature [27].

Both, overall survival and recurrence-free survival were significantly influenced by the three stage groupings in early (I + II), advanced (III), and metastatic (IV) stages. In contrast, a recent study showed only a significant deterioration regarding the recurrence-free survival and not the overall survival [27]. Overall 5-year survival of 73% and recurrence-free survival of 66% for all patients in this study group was encouraging, although approximately 69% of patients had advanced stages III and IV thymoma/thymic carcinoma.

The survival curves in both staging systems showed a significant deterioration of prognosis as the stage increased up to stage III. In contrast, stage IV patients with pleural or lymphatic tumor spread had a significant better overall survival than patients in stage III. This survival benefit might be explained, because almost all our patients in Masaoka-Koga stage IVa underwent successful radical surgical resection, which was also combined with the HITHOC (68%) within the last years [28]. Finally, surgical resection within a multimodality treatment concept in patients with pleural tumor dissemination is feasible and leads to encouraging 5-year survival rates up to 79%. This multimodality treatment regime might not be performed in all patients of the collaborative IASLC/ITMIG database and therefore was not taken into account for the final analysis. In addition, advanced stages were probably underrepresented compared to earlier stage tumors [13].

The recurrence-free survival was nearly similar between stage III and IV tumors. That means the biological behavior (e.g., recurrence) of the tumor seems not to be influenced by the tumor stage. But the consequence of tumor recurrence seems to be different in both stages, since mediastinal recurrence after stage III tumors often directly involves vitally important structures (i.e., great vessels, heart). On the other hand, pleural recurrences usually do not affect vital organs and therefore can often be treated again surgically, which leads to a prolonged survival. These patients might benefit from multimodal treatment including surgery and should be evaluated interdisciplinary [3, 29–31].

## Conclusion

In conclusion, the new TNM-staging system presents a clinically useful and applicable system, which can be used for indication and stage-adapted therapy. It also seems to serve as a potential prognostic prediction model for overall and recurrence-free survival. Advanced stage IVa patients can be treated with multimodality therapy including radical surgery, which results in a longer survival compared to patients with stage III. Systematic lymph node dissection is recommended and has a strong impact on stage distribution (stage IV). We all should perform systematic lymph node dissection, although its impact on adjuvant therapy and prognosis is still controversial. Further studies using prospectively collected data (especially advanced stages and lymph node status) of large patient cohorts on the proposed TNM-staging system are warranted to prove our results and would be helpful for uniform classification of thymic tumors, stage-adapted therapy, and prediction of prognosis.

### Strengths and limitations

We are aware of the potential restrictions of our study. The obvious limitation is its retrospective, non-randomized nature. All data were revalidated by another coauthor before analysis. This procedure should have reduced errors, but did not eliminate them completely. In particular, the sample size is limited. However, we present a cohort of surgical patients with a high percentage of advanced stages, who were treated within a multimodality therapy regime. We have a complete follow-up of all patients; thus, the follow-up period might be relatively short for thymoma, because of the slow progression and late recurrence of this tumor entity.
